# Medicinal Plants Used in Traditional Management of Cancer in Uganda: A Review of Ethnobotanical Surveys, Phytochemistry, and Anticancer Studies

**DOI:** 10.1155/2020/3529081

**Published:** 2020-03-15

**Authors:** Timothy Omara, Ambrose K. Kiprop, Rose C. Ramkat, Jackson Cherutoi, Sarah Kagoya, Decrah Moraa Nyangena, Tsedey Azeze Tebo, Papias Nteziyaremye, Lucy Nyambura Karanja, Abigael Jepchirchir, Alfayo Maiyo, Betty Jematia Kiptui, Immaculate Mbabazi, Caroline Kiwanuka Nakiguli, Brenda Victoria Nakabuye, Margaret Chepkemoi Koske

**Affiliations:** ^1^Department of Chemistry and Biochemistry, School of Biological and Physical Sciences, Moi University, Uasin Gishu County, P.O. Box 3900-30100, Eldoret, Kenya; ^2^Department of Quality Control and Quality Assurance, Product Development Directory, AgroWays Uganda Limited, Plot 34-60, Kyabazinga Way, P. O. Box 1924, Jinja, Uganda; ^3^Africa Center of Excellence II in Phytochemicals, Textiles and Renewable Energy (ACE II PTRE), Moi University, Uasin Gishu County, P.O. Box 3900-30100, Eldoret, Kenya; ^4^Department of Biological Sciences, School of Biological and Physical Sciences, Moi University, Uasin Gishu County, P.O. Box 3900-30100, Eldoret, Kenya; ^5^Department of Chemistry, Faculty of Science, Kyambogo University, P.O. Box 1, Kyambogo, Kampala, Uganda; ^6^Department of Quality Control and Quality Assurance, Product Development Directory, Sweets and Confectionaries Section, Kakira Sugar Limited, P.O. Box 121, Jinja, Uganda; ^7^Southern Agricultural Research Institute (SARI), Hawassa Agricultural Research Center, P.O. Box 2126, Hawassa, Ethiopia; ^8^Chemistry Department, Faculty of Science, Mbarara University of Science and Technology, P.O. Box 1410, Mbarara, Uganda; ^9^Department of Food Processing Technology, Faculty of Science, Kyambogo University, P.O. Box 1, Kyambogo, Kampala, Uganda; ^10^Department of Quality Control and Quality Assurance, Leading Distillers Uganda Limited, P.O. Box 12369, Kampala, Uganda; ^11^Department of Chemistry, Faculty of Science, Egerton University, P.O. Box 536-20115, Njoro, Kenya

## Abstract

The burden of neoplastic diseases is a significant global health challenge accounting for thousands of deaths. In Uganda, about 32,617 cancer cases were reported in 2018, accompanied by 21,829 deaths. In a view to identify some potential anticancer plant candidates for possible drug development, the current study was designed to compile the inventory of plants with reported anticancer activity used in rural Uganda and the evidences supporting their use in cancer therapy. An electronic survey in multidisciplinary databases revealed that 29 plant species belonging to 28 genera distributed among 24 families have been reported to be used in the management of cancer in Uganda. Anticancer plants were majorly from the families Bignoniaceae (7%), Caricaceae (7%), Fabaceae (7%), Moraceae (7%), and Rutaceae (7%). Most species occur in the wild (52%), though some are cultivated (48%). The growth habit of the plants is as trees (55%) or herbs (45%). Anticancer extracts are usually prepared from leaves (29%), bark (24%), roots (21%), and fruits (13%) through decoctions (53%), as food spices (23%) or pounded to produce ointments that are applied topically (10%). *Prunus africana* (Hook.f.) Kalkman, *Opuntia* species, *Albizia coriaria* (Welw. ex Oliver)*, Daucus carota* L., *Cyperus alatus* (Nees) F. Muell., *Markhamia lutea* (Benth.) K. Schum., and *Oxalis corniculata* L. were the most frequently encountered species. As per global reports, *Allium sativum* L., *Annona muricata* L., *Carica papaya* L., *Moringa oleifera* Lam., *Opuntia* species, *Prunus africana* (Hook.f.) Kalkman, and *Catharanthus roseus* (L.) G. Don. are the most studied species, with the latter having vincristine and vinblastine anticancer drugs developed from it. Prostate, cervical, breast, and skin cancers are the top traditionally treated malignancies. There is a need to isolate and evaluate the anticancer potential of the bioactive compounds in the unstudied claimed plants, such as *Cyperus alatus* (Nees) F. Muell., *Ficus dawei* Hutch., *Ficus natalensis* Hochst., and *Lovoa trichilioides* Harms, and elucidate their mechanism of anticancer activity.

## 1. Introduction

Cancer ascribes a collection of diseases triggered by the uncontrolled proliferation of malignant cells. It is a global health burden that has left anintolerable death toll worldwide. Conservative estimates indicate that cancer (of the liver, breasts, lungs, cervix uteri, stomach, and colorectal) causes about 13% of annual deaths globally [1]. In Uganda, there have been reports on cancer cases, though collected data are not usually coherent [2]. The commonest types of cancer encountered in Uganda include cervical, prostate, breast, lung, and skin cancers, Kaposi sarcoma, Burkitt's lymphoma, and cancer of the bone, eye, colon, and blood (leukemia) [3]. Between 1952 and 1953, 796 cases of cancer were reported in Uganda, 15 of which were stage IV cases of cancer of the cervix uteri [4]. The 1990s recorded Kaposi sarcoma, prostate, and oesophageal cancers among men and Kaposi sarcoma, cervical, and breast cancers among women as the most prevalent cancers in Uganda [5].

The eruption of the ill-fated virus (HIV) and the AIDS epidemic led to an unprecedented increase in the incidences of Kaposi sarcoma, squamous cell carcinoma of the conjunctiva, and non-Hodgkin's lymphoma in the penultimate year [6]. Cervical cancer, the fourth most prevalent cancer globally, subsequently registered an alarming relative frequency in Uganda, with 3, 915 new cases and 2, 275 annual deaths reported [7, 8]. The situation is complicated by the fact that very few (averagely less than 10% of the 10.22 million susceptible Ugandan females) have been screened for cervical cancer [7, 9]. Reluctancy to screen, stigma, lack of awareness, chronic poverty, and inadequate medical services are largely responsible for the magnitude of the cancer epidemic in Uganda [9–11]. Thus, most cancer cases registered in hospitals are usually in their advanced stages that cannot be treated optimistically [12, 13].

Cancer trends for two decades (1991–2010) in the Kampala cancer registry have indicated that there has been an increase in cancer cases, peculiarly for breast cancer and prostate cancer in women and men, respectively [14]. On the other hand, the incidence of the oesophagus, liver, and large bowel (colon, rectum, and anus) cancers has remained relatively constant [14]. According to a recent report based on Gulu and Kampala cancer registries [15], 32,617 cancer cases were registered in the country in 2018 and about 21,829 of these victims succumbed to death. In Uganda, the survival of cancer patients is alarmingly deplorable in comparison to other non-African third world nations [2, 16]. The risk factors cited for the high cancer incidences in Uganda include acquisition from family lines, hormonal imbalances, consumption of mycotoxin (aflatoxin) contaminated foods, exposure to chemicals, irradiation, viruses, and bacteria [3, 17–19].

Conventional therapies for the management of cancer have several side effects due to their lack of specificity and are limited in rural settings [20]. Further, the sturdy resistance of cancerous cells to cytotoxic and antineoplastic drugs has presented a fresh challenge, giving unsatisfactory ministration outcomes and capricious resistance to antineoplastic agents [21, 22]. Coupled with the prohibitive costs, unavailability of allopathic drugs, and chronic poverty in Uganda, there is a need to fold back on home grown solutions, exploring flora and fauna [23]. Uganda, the pearl of Africa, is part of the East African botanical block and is blessed with over 6,000 plant species [24, 25]. Plants are regaining shape and emerging as an integral part of the ethnomedical approach for the management of diseases in Uganda [26]. The most cathartically notable anticancer botanical species in Uganda are *Prunus africana* (Hook.f.) Kalkman and the periwinkle plant (*Catharanthus roseus* (L.) G. Don) from which antitumor drugs vinblastine and vincristine have been developed [23, 27]. Over 5,000 phytochemicals such as phenolics, carotenoids, glucosinolates, terpenoids, and alkaloids from over 3,000 plant species have been reported to be key actors in cancer therapy [28–30]. In Uganda, anecdotal reports reveal that there exists a pool of plants utilized locally for the management of cancer [23]. Indeed, information on indigenous medicinal plants used for various maladies has been reported by preceding authors, but none of them scrutinized anticancer plants. This review seeks to obtain a list of medicinal plants reported by ethnobotanical surveys in Uganda as anticancer plants and identify the active phytochemicals in the claimed plants and the anticancer studies done on them as per global studies. Potential candidates from this review which are scantily studied will be investigated in penultimate studies.

## 2. Methodology

A comprehensive literature search was performed in Scopus, Web of Science Core Collection, PubMed, Science Direct, Google Scholar, and Scientific Electronic Library Online (SciELO) from August 2019 to November 2019 following procedures previously employed elsewhere [26]. The search was performed independently in all databases. The study databases included original articles published in peer-reviewed journals, books, thesis, dissertations, patents, and other reports covering anticancer plants, dated until November 2019. All publishing years were considered, and articles with information on cancer or medicinal plants in Uganda were given utmost priority. Thus, references contained within the returned results were assessed concerning their inclusion in this study, and further searches were carried out at the Google search engine using more general search terms, to broaden the search, as follows: words cancer, plants, plant extract, vegetal, vegetal species, vegetal extract, traditional medicine, alternative medicine, complementary therapy, natural medicine, ethnopharmacology, ethnobotany, herbal medicine, herb, herbs, decoction, infusion, macerate, cancerous, hepatocellular carcinoma, carcinoma, prostate cancer, breast cancer, Kaposi sarcoma, Burkitt's lymphoma, cancer of the bone, cancer of the eye, cancer of the colon, cancer of the blood, leukemia, anticancer, cancer of the cervix uteri, lung cancer, liver cancer, skin cancer, and Uganda were used. The last search was done on 25^th^ November 2019. The search outputs were saved where possible on databases and the authors received notification of any new searches meeting the search criteria from Science Direct, Scopus, and Google scholar.

## 3. Results and Discussion

**Figure 1:**
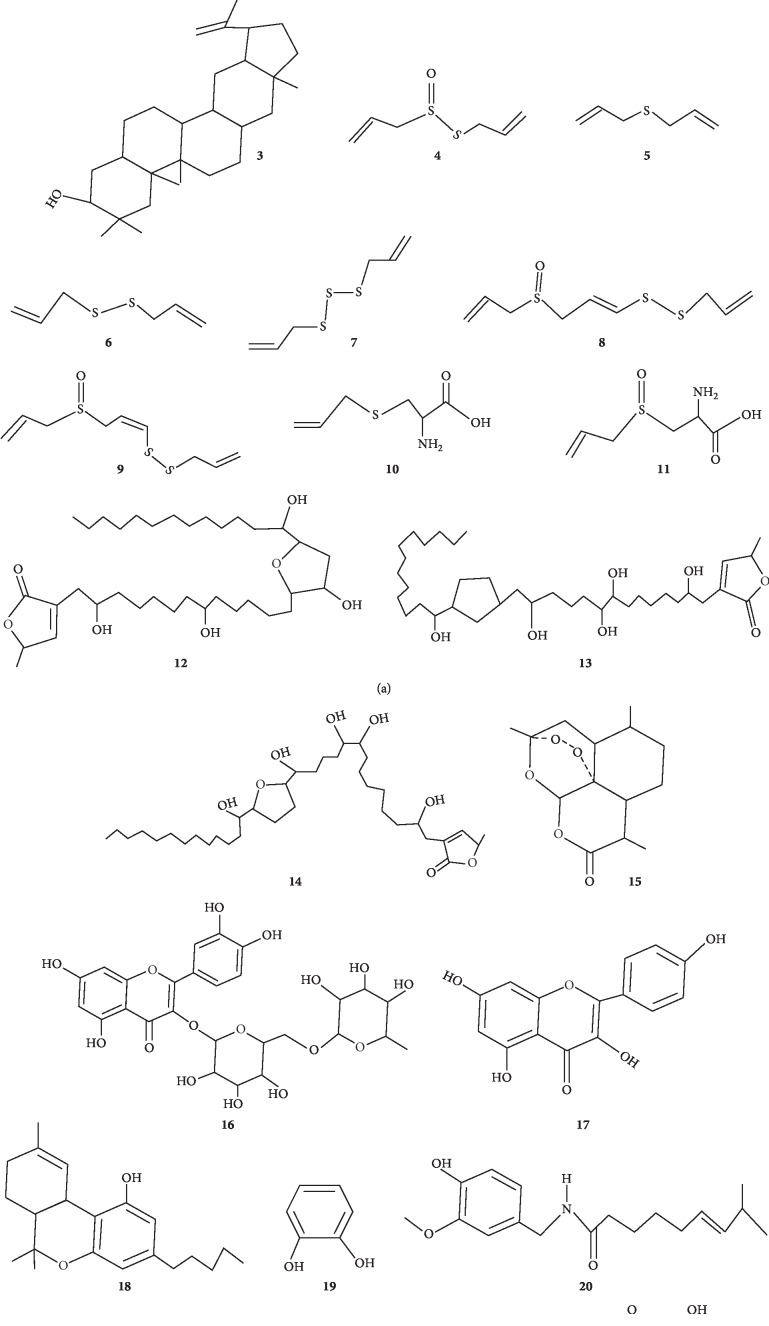
Some of the anticancer molecules reported in some anticancer plants used in rural Uganda. The numbers 1–22 correspond to the molecules mentioned in Table 2.

Only articles in English and local languages were considered. After the multidisciplinary database and Google search engine result assessments, sixteen reports of interest specifically on the subject of anticancer plants in Uganda were retrieved (Table 1). The botanical names of the plants listed were vetted in botanical databases: The Plant List [47], International Plant Names Index (IPNI) [48], NCBI taxonomy browser [49], and Tropicos [50]. Where a given species was considered as distinct species in different reports, the nomenclature as per the botanical databases above took precedence in the review. The botanical families used, the plant local names (Lango, Ateso, Luganda, Rukiga, Rutoro, Lusoga, Lugisu, Ngakarimojong, and Lugbara), the life forms, part(s) used, conservation status, preparation and administration mode, and the districts where the plants were reported are captured (Table 1). On anticancer potential, species studied as per global reports, the active phytochemicals reported and tested with positive results in the plant species identified by this review are reported (Table 2; Figure 1). A brief review of other ethnomedical uses of the reported species as per Ugandan and global studies is also presented (Table 3).

### 3.1. Traditional Concept of Cancer in Uganda

From the electronic survey, it is clear that local communities in Uganda have some information about cancer. Not all Ugandans are fully aware of cancer because most information on it is disseminated through television and radio stations which not all have access to. Another striking challenge is that there is no word for cancer in any of the Ugandan local languages. Thus, many ignore cancer because it is not anywhere recited as a health problem in their local vocabulary [16]. To many, being diagnosed with any type of cancer is equated to receiving a death sentence [234]. Some believe that conventional treatments usually cause cancer to spread, fastening the death of victims [16]. In addition, due to the ever-changing landscape of available treatment options, most patients believe that cancer can only be cured using herbal medicine and the best way to deal with cancers is through prevention [23]. Many Ugandans assume that herbal products are safer to use than allopathic drugs. In Northern Uganda, the use of shea (*Vitellaria nilotica*) butter, simsim (*Sesame indicum* L.), and groundnut (*Arachis hypogea* L.) pastes as substitutes for refined cooking oil and vaseline with the belief that the latter are carcinogenic is known. Unfortunately, recent reports have pointed out that some of these food items are contaminated with mycotoxins, particularly aflatoxins which are potential carcinogens [19, 235].

### 3.2. Anticancer Plants Used in Local Communities of Uganda

Cancer chemoprevention which involves the inhibition or reversion of cancer through the administration of natural or synthetic agents has gained a wider audience in Uganda. Chemopreventive agents may inhibit cancer development either by limiting exposure to carcinogens (carcinogen formation inhibitors and blocking agents) or by decreasing tumor promotion or progression stages (suppressing agents) [236]. Many compounds from medicinal or dietary plants have been reported as chemopreventive agents capable of inhibiting DNA damage and retarding or reversing carcinogenesis in *in vitro* and *in vivo* bioassays [237].

From our survey, 29 plant species from 28 genera belonging to 24 botanical families claimed as anticancer plants in Uganda have been reported (Table 1). The most cited families were Bignoniaceae (7%), Caricaceae (7%), Fabaceae (7%), Moraceae (7%), and Rutaceae (7%). Most families encountered in this study have reported use in the traditional management of cancer in other countries across the globe. For example, Apocynaceae, Asteraceae, Bignoniaceae, Caricaceae, Fabaceae, Malvaceae, Meliaceae, Moraceae, Rutaceae, Sapindaceae, and Solanaceae were cited in Kenya [170], Ethiopia [238], Tanzania [97], and Near East (Arabian Peninsula, Egypt, Iraq, Iran, Israel, Jordan, Lebanon, Palestinian territories, Syria, and Turkey) [239], Lamiaceae in Morocco [240], and Apocynaceae, Meliaceae, and Malvaceae in Sri Lanka [241].

In addition, some of the plant species recapitulated have been documented in the treatment of cancer globally; for example; *Carica papaya* L., *Catharanthus roseus* (L.) G. Don, and *Prunus africana* (Hook.f.) Kalkman were reported to be used for traditional treatment of cervical, colorectal, prostate, and breast cancers [114, 170, 242] while *Albizia coriaria* Welw. ex Oliver, *Capsicum frutescens* L., and *Kigelia africana* (Lam.) Benth. has been reported for the treatment of squamous cell carcinoma, throat, and breast cancers in Kenya [170]. *Zanthoxylum chalybeum* Engl. is used in Ethiopia and Tanzania for the treatment of breast and cervical cancers [97, 243], *Blighia unijugata* Baker is used for the treatment of breast and cervical cancers in Tanzania [97], while *Cymbopogon citratus* (DC.) Stapf is used against colorectal cancer in Kenya [170]. Interestingly, some of these plants are consumed as food spices; for example, *Cymbopogon citratus* (DC.) Stapf is used by most communities in Northern Uganda who cannot afford tea (*Camellia sinensis* (L.) Kuntze) leaves, and *Beta vulgaris* L. and *Daucus carota* L. are common ingredients in Ugandan culinary recipes [93]. Indeed, epidemiological studies have supported that dietary intake of fruits, vegetables, and teas tends to lower the risk of human cancers [244].

Further, some of the botanical species have been reported as recipes of anticancer preparations in other countries. For example, *P. africana* stem bark is used in combination with *Harungana madagascariensis* Lam. ex Poir, *Zanthoxylum gilletii* (De Wild.) P.G. Waterman (stem bark), *Spathodea campanulata* P. Beauv, and *Vernonia lasiopus* O. Hoffman (stem bark), and *P. africana* (stem bark and roots), *Aloe volkensii* leaves, *Spathodea campanulata* P. Beauv (leaves and stem bark), and *Harungana madagascariensis* Lam. ex Poir (stem bark) boiled with *Trichilia emetica* Vahl. are used for the treatment of skin, breast, and colorectal cancers in Kenya [170]. Similarly, *Markhamia lutea* (Benth.) K. Schum stem bark alone or in combination with *Albizia gummifera* stem bark and *Conyza sumatrensis* (Retz.) E.H Walker leaves is used in the management of squamous cell carcinoma of the gums, colorectal, throat, and breast cancers in Kenya [170]. In Tanzania, *Kigelia africana* (Lam) Benth. stem bark mixed with approximately equal weights of root barks of *Maclura africana* (Bureau) corner, *Harrisonia abyssinica* Oliv., and *Warburgia stuhlmannii* Engl. is drunk for the treatment of breast, liver, and colon cancers [97]. *Euclea natalensis* A. DC. root bark boiled with the root barks of *Harrisonia abyssinica* is drunk as a treatment for leukemia in Tanzania [97].

Some of the plants have been reported to have cytotoxic and antitumor properties (Table 2) and many possess other ethnomedical applications (Table 3) both in Uganda and internationally. Interestingly, the isolation, characterization, and purification of the anticancer and cytotoxic phytoconstituents have been successfully done in some species (Figure 1). Striking examples are *Prunus africana* (Hook.f.) Kalkman which have been patented in France for the management of prostate cancer [245] and *Catharanthus roseus* (L.) G. Don from which the commercial anticancer drugs vincristine and vinblastine have been developed [246].

Phytochemicals from plants are reported to be effective against cancer cells because they have many molecular targets [247]. For example, *β*-sitosterol present in *P. africana* has been shown to exhibit anti-inflammatory, antineoplastic, and immunomodulating activities [248]. It is worth mentioning that antioxidant activities and antitumor or anticancer properties of plant extracts are always reported concomitantly in several plants [166], and some studies demonstrated that there is a positive linear relationship between antioxidant activity and anticancer effect of plant extracts [249]. Plant phytochemicals such as artemisinin from the *Artemisia* genus are reported to have an endoperoxide moiety which is strategic for their bioactivity. The cleavage of this is reported to produce reactive oxygen species, inducing oxidative stress. Furthermore, in the presence of ferrous iron or reduced heme, artemisinin can convert itself into cytotoxic carbon-centred radical, a highly potent alkylating agent, to induce direct oxidative damage to cancer cells [250, 251]. Thus, they are reported to induce apoptosis and ferroptosis, reduce cell proliferation through cell cycle arrest, and inhibit angiogenesis and tissue invasion of the tumor as well as cancer metastasis [184, 250, 251].

### 3.3. Growth Habit, Parts Used, Preparation, and Mode of Administration

Most anticancer plant species reported in Uganda occur in the wild (52%) though some are cultivated (48%). The growth habit of the plants is as trees (55%) or herbs (45%). Anticancer extracts are usually prepared from leaves (29%), bark (24%), roots (21%), fruits (13%), seeds (5%), bulb (5%), or rhizomes (3%). The regular use of roots and leaves in traditional medicine is a characteristic feature of *materia medica* in Uganda [26]. As reported elsewhere [238, 240], embryonal plant parts such as fruits, seeds, buds, bulbs, and flowers which are reported to accumulate bioactive compounds are less frequently used in anticancer therapy in Uganda.

Usually, anticancer preparations are presented as decoctions and teas (53%) and spices eaten in food (23%) or pounded to produce ointments that are applied topically (10%). The plants are collected from the wild, cultivated fields, or home gardens when needed. Traditional medicine practitioners either collect herbal plants personally or hire collectors. All traditional medical practitioners cultivate some medicinal plants especially fast growing ones around their homes and shrines in order to have them within easy access when needed [26]. The preparations are majorly administered orally, except in cases of skin cancers where they are applied topically as ointments.

### 3.4. Other Ethnomedicinal Uses and Toxicity of the Reported Anticancer Plants

Almost all the plants recapitulated in this review are employed for the treatment of various ailments other than cancer. *Albizia coriaria* (Welw. ex) Oliver used in the management of venereal diseases (syphilis, HIV, and gonorrhoea), postpartum haemorrhage, sore throats, menorrhagia, threatened abortion, skin diseases, jaundice, cough, and sore eyes [33, 179] is a good representative example. Such plants tend to be used in different communities for treating cancer and can be a good justification for their pharmacological efficacy [26].

On the other hand, some of the anticancer plants cited exhibit marked toxicity. A striking example is *Catharanthus roseus* (L.) G. Don. The alkaloids in it are neurotoxic, especially vincristine [252]. Vincristine and vinblastine are highly toxic antimitotics, blocking mitosis in metaphase after binding to the microtubules [253]. Evidently, side effects such as myelosuppression, alopecia, abdominal cramps, constipation, nausea, paralytic ileus, ulcerations of the mouth, hepatocellular damage, kidney impairment, pulmonary fibrosis, urinary retention, amenorrhoea, azoospermia, orthostatic hypotension, and hypertension [254–256] have been reported for the commercial drugs vincristine and vinblastine derived from this plant. In essence, the administration of these drugs must be carefully controlled to reduce the side effects [257]. This observation explains, in part, why some anticancer preparations in Uganda are applied topically or ingested in small amounts. Fortuitously, topical application is a better approach for reducing the local action of cancer cells at externally affected parts.

### 3.5. Clinical Studies

At present, clinical trials utilizing standardized extracts of anticancer plants reported in Uganda or their bioactive compounds have not been done with the exception of *Prunus africana* (Hook.f.) Kalkman and *Catharanthus roseus* (L.) G. Don. which have been investigated in other countries [245, 246]. Prostafx, Tadenan, and Pygenil are some of the herbal preparations of *Prunus africana* (Hook.f.) Kalkman on the market. Due to the paucity of data generated from preclinical tests (pharmacokinetic and toxicological studies) and the regulatory requirements for clinical studies, the safety and efficacy of traditional anticancer plant preparations used in Uganda remain a secret yet to be unveiled. Although there are many research institutes such as Uganda Virus Research Institute, Natural Chemotherapeutic Research Institute, Uganda Industrial Research Institute, and National Agricultural Research Institute, none is designed to have an in-depth focus on drug discovery and development to the level of commercialization. Thus, the government of Uganda should establish an institute that handles drug discovery and development to enhance the utilization of medicinal plants in Uganda.

## 4. Conclusions and Recommendations

The inventory of plants utilized by Ugandan communities presents considerable potential for the treatment of cancer. *Cyperus alatus* (Nees) F. Muell, *Ficus dawei* Hutch*, Ficus natalensis* Hochst, and *Lovoa trichilioides* Harms are some of the plants with claimed anticancer potential that have been hardly studied and therefore warrant further investigations. More ethnobotanical surveys should be done in the unsurveyed districts to identify other potential anticancer plants. *Albizia coriaria* Welw. ex Oliver which doubles as an antivenin plant will be investigated for its anticancer potential in a penultimate study.

## Figures and Tables

**Table 1:** Plants used in the management of cancer in rural Uganda as per reports of ethnobotanical surveys.

Plant family	Local name	Botanical name	Part used	Life form	Conservation status	Mode of preparation (administration)	Cancer treated	District (s)	Author (s)
Amaranthaceae	Beetroot (*Eng*), no local name	*Beta vulgaris* L.	Blb	H	C, NE	Eat beetroot/prepare juice and drink	Blood cancer	Kampala, not specified	[31, 32]
Amaryllidaceae	Garlic (*Eng*), katungulusumu (*Lug)*	*Allium sativum* L.	Blb	H	C, NE	Chew/eat regularly or put in food	Lung cancer	Kampala	[31]
Annonaceae	Kitafeeri (*Lug*) Obwolo (*Lang*)	*Annona muricata* L.	R, L, F	T	C, NE	Decoction drunk	Not specified	NS	[33]
Apiaceae	Carrot (*Eng*), no local name	*Daucus carota* L.	R	H	C, NE	Eat raw roots regularly; used with beetroot	Blood cancer	Wakiso, Kampala	[31, 34]
Apocynaceae	Sekagya (*Lug*)	*Catharanthus roseus* (L.) G. Don	F	H	C, NE	Not specified	Not specified	Kampala	[31]
Asteraceae	Artemesia (*Eng*), no local name	*Artemisia annua L.*	L	H	W, NK	Infusion with rock salt	Not specified	NS	[33]
Bignoniaceae	Yago (*Lang, Acholi*), Edodoi (*Ateso*), Sifungu, Omusa (*Lug*), Naizungwe (*Lus*)	*Kigelia africana* Lam. Benth.	R, B	T	W, NK	Crushed in water to make a concoction; 1-2 tablespoonfuls of juice taken orally twice a day	Not specified	Tororo/Mbale	[35, 36]
Bignoniaceae	Sambya (*Lug*), Lusambya (*Lus*), Lusoola (*Gis*), Musambia (*Rut, Ruk*)	*Markhamia lutea* (Benth.) K. Schum.	F	T	W, NK	Not specified	Not specified	Not specified	[37, 38]
Burseraceae	Mwafu (*Lug*), Mubafu (*Lus, Rut*)	*Canarium schweinfurthii* Engl.	F	T	C, NE	Eat fruits	Not specified	Not specified	[39]
Cactaceae	Prickly pear cactus (*Eng*)	*Opuntia* species^*∗*^	L	H	C, NE	Take the juice from leaves	Prostate, stomach, colon and rectum cancer	Kampala	[31, 40, 41]
Capparaceae	Njagga (*Lug*), Enjaaye, Njagga (*Rut*), Jai (*Lang)*	*Cannabis sativa* L.	L	H	C, NE	Decoction drunk	Not specified	NS	[33]
Caricaceae	Mapapali (*Lug*), apapalo (*Lang*), papali (*Ateso*), Paipai (*Lgb*)	*Carica papaya* L.	L	H	C, NE	Decoction drunk	Not specified	Pallisa	[42]
Caricaceae	Not reported	*Euclea natalensis* A.DC.	B	T	W, NK	Decoction drunk	Prostate cancer	Mukono/Buikwe	[43]
Cyperaceae	Not reported	*Cyperus alatus* (Nees) F. Muell	RZ	H	W, NK	Not specified	Not specified	Not specified	[37, 38]
Fabaceae	Omugavu (*Lug*), Musiita (*Lus*), Kiluku (*Kar*), Itek (*Lang*), Ober (*Acholi*)	*Albizia coriaria* (Welw. Ex) Oliver	B	T	W, C, NE	Decoction drunk/applied as an ointment	Not specified	NS	[33]
Fabaceae	Jjirikiti (*Lug*), Murinzi, Kiko Omoko/Echuko, (*Rut/Ruk*), Oluo (*Lgb*), Owila kot (*Lang*), Muyirikiti (*Lus*)	*Erythrina abyssinica* Lam. ex DC.	B, R	T	W, NK	Decoction drunk	Not specified	NS	[33]
Lamiaceae	Mubengeya, Nfulubwa, Ffulubwa (*Lug*)	*Vitex fischeri* Gürke	L	T	W, NK	Decoction drunk	Not specified	NS	[33]
Malvaceae	Okra (*Eng*), Otigo (*Lang*), Bamia (*Lus*)	*Abelmoschus esculentus* (L.) Moench	F	H	C, NE	Eat as food or add to food as a spice	Stomach, rectum and colon cancer	Kampala	[31]
Meliaceae	Musonko (*Lug*)	*Lovoa trichilioides* Harms	B, Sd, L	T	W, E	Crushed and applied as an ointment	Not specified	NS	[33]
Moraceae	Muwo (*Lug*)	*Ficus dawei* Hutch.	B	T	W, NK	Decoction drunk	Breast cancer	Mukono/Buikwe	[43]
Moraceae	*Mugaire (Lus)*	*Ficus natalensis* Hochst.	R	T	W, NE	Not specified	Cancerous wounds	Iganga	[44]
Moringaceae	Moringa (*Eng*), molinga (*Lug*)	*Moringa oleifera* Lam.	L, R, B, Sd	H	C, NE	Chew/make juices	Prostate, lung, colon and rectal cancers	Kampala	[31, 45]
Oxalidaceae	Kajjampuni (*Lug*), *Kaanhunu (Lus),* Otyer (*Lang*)	*Oxalis corniculata* L.	L	H	W, E	Pound, dry and put on the wound	Skin and uterine cancer	Mukono/Buikwe, Iganga	[43, 44]
Poaceae	Akisube (*Ateso*), Kisubi (*Lug*), Lum cai (*Lang*)	*Cymbopogon citratus* (DC) Stapf	L	H	C, NE	Decoction drunk	Not specified	Pallisa	[42]
Rosaceae	African cherry (*Eng*), Ngwabuzito (*Lug*), Ntaseesa, Ngwabuzito (*Rut*), Sirumandu (*Gis*)	*Prunus africana* (Hook.f.) Kalkman (*Pygeum africanum*)	L, B	T	W, OD	Decoction drunk/tea	Prostate cancer	NS, Not specified, Mukono/Buikwe, Not specified	[23, 33, 43, 46]
Rutaceae	Omuqugwa (*Ateso*), Amacunga (*Lang)*	*Citrus reticulata* Blanco, 1837	R	T	C, NE	Not specified	Not specified	Pallisa	[42]
Rutaceae	Ntale ya ddungu (*Lug*), Eusuk (*Ateso*), Agodaman (*Lang*), Rukuts (*Kar*), Outiku (*Lugb*)	*Zanthoxylum chalybeum* Engl.	R	T	W, NK	Pound, add water & drink	Cervical cancer	Mukono/Buikwe	[43]
Sapindaceae	Mukuzanyana (*Lug*), Nkuzanyana (*Rut*)	*Blighia unijugata* Baker	B	T	W, NK	Decoction drunk	Cervical cancer	Mukono/Buikwe	[43]
Solanaceae	Kamulali (*Lug*), Kamularu (*Lang*)	*Capsicum frutescens* L.	F	H	W, C	Food condiment	Prostate cancer	Mukono/Buikwe	[43]

Languages: *Eng: English, Gis*: *Lugishu*, Lgb*: Lugbara, Lug: Luganda, Lus: Lusoga, Lang: Lango, Kar: Ngakarimojong, Rut: Rutoro, Ruk: Rukiga*. Parts used: B: bark, Blb: bulb, F: fruit, L: leaf, R: root, RZ: rhizome, Sd: seed. Growth habit: H: herb, T: tree. Conservation status: C: cultivated, W: grows in the wild/forest, E: endangered, NE: not endangered, OD: out of danger, NK: not known. Districts: NS: this study was done in Arua, Dokolo, Mbale, Iganga, Bushenyi, Rakai, Luwero, and Kaabong districts of Uganda. ^*∗*^Species not specified. *Opuntia* hybridizes readily between species.

**Table 2:** Anticancer activity of the medicinal plant species reported in Uganda as per global reports.

Plant	Active phytochemicals	Molecular targets and/or effects on cancer cells
*Abelmoschus esculentus* (L.) Moench	Isoquercitrin (**1**), quercetin (**2**), hyperoside (hyperin), coumarin scopoletin, and uridine [51]	Cytotoxicity of extracts reported against breast cancer (MCF-7), hepatocellular carcinoma (HepG2), and cervical carcinoma (HeLa) cell lines [51]. Isoquercitrin inhibited urinary bladder, pancreatic, and colon cancer progress [52–54]
		Extracts induced significant cell growth inhibition (63%) in human breast cancer (MCF-7) and skin fibroblast (CCD-1059 sk) cells. The expression of proapoptotic caspase-3, caspase-9, and p21 genes was increased in MCF-7 cells [55]

*Albizia coriaria* (Welw. ex Oliver)	Oleanane-type saponins (coriarioside A and coriarioside B), gummiferaoside C, acacic acid glycosides, lupeol (**3**), lupenone, betulinic acid, acacic acid lactone, (+) – catechin, and benzyl alcohol [56–58]	Cytotoxicity (IC_50_ > 500 *μ*g/ml) against human embryonic lung fibroblast (HELF) cells [59]. Coriarioside A and gummiferaoside C from root bark showed cytotoxicity against two colorectal human cancer cells: HCT116 (with IC_50_ of 4.2 *μ*M for coriarioside A and 2.7 *μ*M for gummiferaoside C) and HT-29 (with IC_50_ 6.7 *μ*M for coriarioside A and 7.9 *μ*M for gummiferaoside C) cell lines [56]

*Allium sativum* L.	Diallyl thiosulfinate (allicin) (**4**), diallyl sulfide (DAS) (**5**), diallyl disulfide (DADS) (**6**), diallyl trisulfide (DATS) (**7**), E-ajoene (**8**), Z-ajoene (**9**), S-allyl-cysteine (SAC) (**10**), and S-allyl-cysteine sulfoxide (alliin) (**11**) [60]	Extracts exhibited an antiproliferative effect on human cancer cell lines, including liver (HepG2), colon (Caco2), prostate (PC-3), and breast (MCF-7) cancer cells [61]. Extracts induced G2/M-phase cell cycle arrest in EJ bladder cancer cells [62]. DATS suppressed the proliferation of SGC-7901 gastric cancer cells [63]
		SAC induced cell cycle arrest in A2780 human epithelial ovarian cancer cells [64]. S-propargyl-l-cysteine (SPRC), an analogue of SAC, reduced the proliferation of human pancreatic ductal adenocarcinoma cells and induced cell cycle arrest [65]. Garlic derived S-allylmercaptocysteine (SAMC) suppressed the proliferation of hepatocellular carcinoma cells [66]. SAMC inhibited the proliferation of human colorectal carcinoma SW620 cells [67]
		Allicin inhibited the proliferation of gastric adenocarcinoma cells by inducing cell cycle arrest [68]. Ajoene was shown to restrain the growth of glioblastoma multiforme cancer stem cells and human breast cancer cells [69]

*Annona muricata* L.	Annonaceous acetogenins (muricin J, muricin K, muricin L) [70], annonacin (**12**), annomuricin A (**13**), annomuricin E (**14**), annomuricin C, annomutacin, gigantetronin [71, 72], quercetin, luteolin 3′7-di-o-glucoside, gallic acid, apigenin-6-c-glucoside, taxifolin (+) [73]	Annonaceous acetogenins exhibited antiproliferative activity against human prostate cancer PC-3 cells [70]. Fruit extracts are cytotoxic against U937 histiocytic lymphoma cell lines with IC_50_ of 10.5, 18.2, and 60.9 *μ*g/ml for ethyl acetate, hexane, and methanol extracts respectively [74]
		Annonacin caused complete suppression of 7,12-dimethylbenz[a]anthracene (DMBA) induced and 12-0- tetradecaboylphorbol-13-acetate (TPA) promoted skin tumorigenesis in mice [75]
		At 0.1 *μ*M, annonacin induced growth arrest and apoptosis in breast cancer (MCF-7) cells [76]
		Annomuricin E was cytotoxic to HT-29 colon carcinoma and CCD841 normal colon cell lines with IC_50_ values of 5.72, 3.49, and 1.62 *μ*g/mL for HT-29 cells at time intervals of 12, 24, and 48 hours of administration, respectively [77]
		Stem extracts suppressed the expression of molecules associated with hypoxia and glycolysis in CD18/HPAF (pancreatic) cancer cells (IC_50_ of 73.0 *μ*g/mL) [78]
		Aqueous leaf extracts exhibited anticancer activity with IC_50_ values of 220, 350, and 250 *μ*g/mL for breast cancer cell lines: MCF7, MDA-MB231, and 4T1, respectively [79]. Leaf extracts recorded cytotoxicity against human bladder cancer (K562) and leukemia cancer (ECV304) cell lines [80]
		Cytotoxicity recorded against Raji cells with IC_50_ values of 90.6, 407.2, and 260.2 *μ*g/mL. Cytotoxic effect of chloroform and n-hexane extracts on HeLa cell lines gave IC_50_ values of 127.3 and 169.2 *μ*g/mL, respectively [81]
		Leaf extracts inhibited cell proliferation in pancreatic cancer cells (capan-1) [82]
		Ethanol extract of seeds showed a cytotoxic effect on MDBK and HEp-2 cells (IC_50_ values:34.5 and 55 mg/mL, respectively) at 24 h, and an IC_50_ value of 49.6 × 10^−3^ mg/mL toward HEp-2 cells at 72 h [83]
		Cytotoxic against kidney epithelial (VERO), stomach cancer (C-678), and human large lung cell carcinoma (H-460) cell lines with IC_50_ values lower than 0.00022 mg/mL for all the cell lines [84]. Cytotoxicity was reported against histiocytic lymphoma cell lines (U937), pancreatic cancer cells (FG/COLO357), breast cancer cells (MDA-MB-435S), immortalized human keratinocytes (HaCat), normal human liver cells (WRL-68), and human skin malignant melanoma (A375) [73, 78, 85–87]. In histiocytic lymphoma cell lines, the extract had an IC_50_ value of 7.8 *μ*g/mL. Toxicity toward FG/COLO357 with an IC_50_ value of 200 *μ*g/mL [78]. Cytotoxic effect of *n*-butanolic extract of leaves against MDA-MB-435S (human breast carcinoma), HaCaT (human immortalized keratinocyte), and WRL-68 (normal human hepatic) cell lines with IC_50_ values of 29.2, 30.1, and 52.4 *μ*g/mL, respectively [73]
		Ethanol extracts of leaves cytotoxic to Ehrlich Ascites carcinoma (EACC) and breast cancer (MDA and SKBR3) cell lines with IC_50_ values of 335.85, 248.77, and 202.33 *μ*g/mL [88]. Fruit extracts had substantial repression of breast cancer cells (MDA-MB-468) growth as well as selective suppression of epidermal growth factor receptor (EGFR) with IC_50_ of 4.8 *μ*g/mL [89]
*Artemisia annua* L.	Sesquiterpene trioxane lactone (artemisinin) (**15**) [90], Chrysosplenol D, arteannuin B, casticin, arteannuic acid, or 6,7-dimethoxycoumarin [91]	Acetonitrile extract inhibited the viability of breast (MDA-MB-231 and MCF-7), pancreas (MIA PaCa-2), prostate (PC-3), and non-small-cell lung cancer (A459) cells. The extracts inhibited cancer cell proliferation, decreased tumor growth, and induced apoptosis *in vivo* in triple negative breast cancer (MDA-MB-231) xenografts grown on the chick chorioallantoic membrane (CAM) assay as well as in nude mice [91]. Hydroalcoholic extract had a cytotoxic effect in a dose-dependent manner against D-17 canine osteosarcoma cell lines better than pure artemisinin, indicating a possible synergistic effect of the phytocomplex and a mechanism of action involving iron and possibly ferroptosis [92]

*Beta vulgaris* L.	Lutein, *β*-carotene, betalains, betaine, ferulic, caffeic, oleanolic, *p*-coumaric and syringic acids, rutin (**16**), kaempferol (**17**), rhamnetin, rhamnocitrin, and astragalin [93, 94]	The ethanolic extract exhibited significant anticancer activity against lung cancer (A549) cell line but only a slight effect against colorectal adenocarcinoma (Caco-2) cell line at 800 *μ*g/mL [94]. Cytotoxicity against PC-3 cells led to a decrease in the growth rate of the cells (3.7% in 3 days) at 29 *μ*g/mL. Comparative cytotoxicity tests in normal human skin (FC) and liver (HC) cell lines showed that the extracts were cytotoxic on the cells, though the activity was lower than that of doxorubicin (8.6% compared to 100%, respectively, at 29 *μ*g/mL concentration in a 3-day test period) [95]

*Blighia unijugata* Baker	Methyl salicylate, oleic acid, 2-morpholinophenazine, octadec-9-enoic acid, 2-[(tert-butyldimethylsily)oxy]-1-isopropyl-dimethyl-benzene, octadecanoic acid, 1,3-dibromo-4,5-dimethylbenzene, 3,7-dimethyl-8-oxo-5-dioxa-spiro [5]-3-methyl-undecanoate, 1,2-bis-(trimethylsilyl)benzene, and octadecanoic acid [96]	Leaves and stem bark extracts had LC_50_ of 539.6 and 389.8 *μ*g/mL in brine shrimp toxicity [97]

*Cannabis sativa* L.	Δ^9^-tetrahydrocannabinol, THC (**18**) [98]	THC and other cannabinoids exhibited antitumor effects in animal models of cancer [99]. The acetone extract exhibited more anticancer activity against breast adenocarcinoma (MCF-7), the glioblastoma (SF-268), and the colon adenocarcinoma (HT-29) cells [100]

*Canarium schweinfurthii* Engl.	3a-Hydroxytirucalla-8, 24-dien-21-oic, 3*α*-hydroxytirucalla-7,24-dien-21-oic and 3*β*-fluorotirucalla-7,24-dien-21-oic acids [101], catechol (**19**), tyrosol, vanillic, and phloretic acids, limonene, phellandrenes, p-hydroxybenzaldehyde, dihydroxyphenylacetic acid, *p*-hydroxybenzoic acid, dihydroxybenzoic acid, pinoresinol, secoisolariciresinol [102], schweinfurthinol, coniferaldehyde, *p*-hydroxycinnamaldehyde, ligballinol, and amentoflavone [103]	Cytotoxicity test on leukemia (CCRF-CEM) cells at 40 *μ*g/mL showed that leaves and bark extracts induced more than 50% growth of this cell line. Fruit mesocarp oil extract and seed kernel oil extracts are expected to be vital for chemoprevention of cancers and other oxidative damage-induced diseases [104]

*Capsicum frutescens* L.	Capsaicin (**20**) and quercetin [105]	Aqueous fruit extracts exhibited anticancer activity (though lower than capsaicin standard) when tested against prostate (PC-3) and breast (MCF-7) cancer cell lines *in vitro* [105]

*Carica papaya* L.	Lycopene, ferulic acid, benzyl isothiocyanate, kaempferol, quercetin, chlorogenic acid, caffeic acid, beta carotene, and *p-*coumaric acid [106, 107]	Pure lycopene and papaya juice inhibited the viability of liver cancer (HepG2) cell line with IC_50_ of 22.8 *μ*g/mL and 20 mg/mL [108]. *n*-hexane seed extract dose-dependently inhibited superoxide generation (IC_50_ = 10 *μ*g/mL) and the viability of acute promyelocytic leukemia (HL-60) cells (IC_50_ = 20 *μ*g/mL), comparable to that of pure benzyl isothiocyanate [109]
		Aqueous extract of flesh (0.01–4% v/v) inhibited the proliferation of breast cancer cell line (MCF-7) [110]. Ethanolic extract of the pericarp (50–640 *μ*g/mL) inhibited the growth of breast cancer cell line (MCF-7) treated with sodium nitroprusside, a nitric oxide donor [111]. Breast cancer cell line (T47D) was inhibited by leaf protein fraction with IC_50_ = 2.8 mg/mL) and induced apoptosis by regulation of protein expression [112]
		Aqueous extracts of leaves (1.25–27 mg/mL) exhibited a concentration-dependent anticancer effect on stomach cancer cell line (AGS), pancreatic cancer cell line (Capan-1), colon cancer cell line (DLD-1), ovarian cancer cell line (Dov-13), lymphoma cell line (Karpas), breast cancer cell line (MCF-7), Neuroblastoma cell line (T98G), uterine cancer cell line (HeLa), and T-cell leukemia cell line (CD26 negative or negative Jurkat) cell lines and suppressed DNA synthesis by suppressing the incorporation of 3H-thymidine [113]
		Aqueous extract of leaves (0.625–20 mg/mL) inhibited the proliferative responses of both haematopoietic and solid tumor cell lines (T-cell lines, H9, Jurkat, Molt-4, CCRF-CEM, and HPB-ALL), Burkitt's lymphoma cell lines (Ramos and Raji), chronic myelogenous leukemia cell line (K562), cervical carcinoma cell line (HeLa), hepatocellular carcinoma cell lines (HepG2 and Huh-7), lung adenocarcinoma cell line (PC-14), pancreatic epithelioid carcinoma cell line (Panc-1), mesothelioma cell lines (H2452, H226, and MESO-4), plasma cell leukemia cell line (ARH77), anaplastic large cell lymphoma cell line (Karpas-299), breast adenocarcinoma cell line (MCF-7), mesothelioma cell line (JMN), and pancreatic adenocarcinoma cell line (Capan-1). In peripheral blood mononuclear cells, the extract reduced the production of IL-2 and IL-4 whereas it increased the production of Th1 types cytokines such as IL-12p40, IL-12p70, INF-*γ*, and TNF-∝. The expression of 23 immunomodulatory genes was enhanced by the addition of papaya extract [114]
		Leaf juice not only exhibited a stronger cytotoxic effect on human squamous cell carcinoma (SCC25 cancer) cells but also produced a significant cancer-selective effect as shown by tests on noncancerous human keratinocyte HaCaT cells [115]

*Catharanthus roseus* (L.) G. Don.	Antitumor terpenoid indole alkaloids: vincristine and vinblastine, serpentine, catharanthine, ajmalicine, akuammine, lochnerine, lochnericine, tetrahydroalstonine, 3′,4′-anhydrovinblastine, serpentine, vincaleukoblastine, leurocristine, vincaleurocristine, vincarodine, vincoline, leurocolombine, viramidine, vincathicine, vincubine, isositsirikine, vincolidine, catharanthine, vindoline, tetrahydroalstonine, vindolinine, reserpine, coronaridine, 11-methoxy tabersonine, tetrahydroalstonine, vindorosidine, hydroxytyrosol, ferulic acid, chlorogenic acid, kaempferol, trisaccharides, quercetin, and petunidin 3-O-(6-O-*p*-coumaroyl) [116, 117]	Cytotoxicity with LC_50_ of 6.7 *μ*g/ml in brine shrimp assay [97, 118, 119]
		Vindoline from leaf extracts was cytotoxic to HCT-116 colorectal carcinoma cell line at 200 *μ*g/mL [120]
*Citrus reticulata* Blanco, 1837	Limonin, isolimonexic acid methyl ether, ichangin, deacetylnomilin, and obacunone [121]	*In vitro* tumor cytotoxicity of different varieties against gastric cancer cell lines (SGC-7901, BGC-823, and AGS) recorded IC_50_ ranging from 20.49 to 368.40 *μ*g/mL [122]. Antiangiogenic effect was reported [123]

*Cymbopogon citratus* (DC) Stapf	Citral (neral and geranial), geraniol and *β*-myrcene, 6-methyl-5-hepten-2-one, and undeca-2-one [124]. Oils contain geranial, neral, myrcene, and beta-pinene [125]	Essential oil exhibited protective action against *N*-butyl-*N*-(4-hydroxibuthyl)nitrosamine-induced DNA damage and a potential anticarcinogenic activity against mammary carcinogenesis in 7,12-dimethylbenz(a) anthracene, 1,2-dimethylhydrazine initiated female Balb/C mice [124]. In synergy with *Taraxacum officinale* root extract induced apoptosis in prostate cancer cells (DU-145 and PC-3). The lowest combination dosage of taxol treatment (0.01 μM with 0.01 mg/mL extract) showed comparable induction of apoptosis to the highest individual treatment dosage of taxol (0.5 *μ*M) [20]

*Daucus carota* L.	*α*-Longipinene, methyl isoeugenol, elemicin, *β*-Selinene, *β*-asarone, *β*-himachalene, *β*-bisabolene, *α*-humulene, widdrol, allo-aromadendrene, *α*-caryophyllene, *β*-caryophyllene, caryophyllene oxide, aromadendrene [126], 2-himachalen-6-ol, (+)-a-longipinene, longicyclene, and *β*-caryophyllene [127]	Chemopreventive against 7,12-dimethyl Benz(a)anthracene-induced squamous cell carcinoma in mice [126]. The aqueous extract has anticancer activity against human promyelocytic leukemia HL-60 cells. Oil extract is chemopreventive against induced skin cancer [128]. Apoptosis was recorded with colon and breast human cancer cell lines; pentane oil fraction showed a cytotoxic effect on human breast adenocarcinoma cell lines MDA-MB-231 and MCF-7, causing cell cycle arrest and increased apoptosis mediated through the Erk pathway [129]

*Elaeodendron buchananii* (Loes.)	Steroidal glycosides, eudesmane-type sesquiterpenoid, and dammarane triterpenoids: elabunin (**21**) [130–132]	Elabunin exhibited moderate cytotoxic activity with a median effective dose (ED_50_) of 100 *μ*g/mL against L- 1210 leukemic cells [132]

*Erythrina abyssinica* Lam. ex DC.	Erythrina alkaloids: erythraline, erysodine, erysotrine, 8-oxoerythraline, and 11-methoxyerysodine [133]	Cytotoxicity with LC_50_ value > 240 μg/ml [134]. *In vitro* cytotoxicity of the crude alkaloidal fraction reported against HeLa, Hep-G2, HEP-2, HCT-116, MCF-7, and HFB4 cell lines with IC_50_ values of 13.8, 10.1, 8.16, 13.9, 11.4, and 12.2 *μ*g/mL [133]

*Euclea natalensis A.DC. *	Mamegakinone, diospyrin (**22**) and 7-methyljuglone from root bark, lupeol, *β* -sitosterol, 20(29)-lupene-3-isoferulate, Isodiospyrin, 5-hydroxy-4-methoxy-2-nathaldehyde, 80-hydroxydiospyrin, neodiospyrin, methylnaphthazarin, euclanone, octahydroeuclein, shinanolone, diospyrin, and natalenone [135–137]	Cytotoxicity of ethanolic roots and stem extracts reported in brine shrimp lethality test [138]
		Cytotoxicity of crude chloroform extract of the roots, diospyrin, and 7-methyljuglone reported against green monkey kidney cells (VERO) and a mouse macrophage cell line, J774A.1. Crude extract and diospyrin had IC_50_ values of 64.87 and 17.78 *μ*g/mL while 7-methyljuglone effected a 90% reduction of growth of Mycobacterium tuberculosis Erdman within J774.1 macrophage at 0.57 *μ*g/mL [139]
		Cytotoxicity of 7-methyljuglone from the root and a series of its derivatives on MCF-7, HeLa, SNO, and DU 145 human cancer cell lines had IC_50_ values ranging from 5.3 to 10.1 *μ*M [135]

*Kigelia africana* Lam. Benth.	Lapachol, 3-(2′- hydroxyethyl)-5-(2″-hydroxypropyl) dihydrofuran-2-(3H)one, specioside, verminoside, and minecoside, kigelin, *β*-sitosterol, 1,3-dimethylkigelin, and ferulic acid	Seed oil suppressed human colon adenocarcinoma (Caco-2) and human embryonic kidney (HEK-293) cell growth in a dose-dependent manner [140]
		An 80% methanol extract of the roots exhibited cytotoxicity to brine shrimps with LC_50_ of 7.2 *μ*g/ml [138]
		Fruit extracts increased the sub-G1 phase (apoptosis) population in HCT116 human colon cancer cells [141]

*Markhamia lutea* (Benth.) K. Schum	Cycloartane triterpenoids, musambins A–C and their 3-Oxyloside derivatives musambiosides A–C [142], Oleanolic acid, pomolic acid, 2-epi-tormentic acid, musambin A, and b-sitosterol-3-O-b-D-glucopyranoside [143, 144]	Anticancer activity against Ehrlich ascites carcinoma cells with an IC_50_ value of 27.0 *μ*g/mL [143]. Cytotoxicity against KB (mouth epidermoid carcinoma) and the human diploid embryonic lung cells (MRC5) though most IC_50_ values were >50 *μ*g/mL [144]

*Moringa oleifera* Lam	Quercetin, kaempferol, *β*-D-glucopyranoside tetradecanoate, *β*-sitosterol, *β*-sitosterol glucoside [145], isothiocyanate, hexadecanoic acid, and eugenol [146]	Cytotoxic against colon cancer (Colo-320 DM), breast cancer (MCF-7), ovary cancer (PA-1), and oral cancer (KB-403) cell lines with IC_90_ value of 3.98, 17.60,12.86, and 8.40 *μ*g/mL, respectively [145]. Methanol extracts were cytotoxic to human B-lymphocyte plasmacytoma (U266B1) cell line with IC_50_ of 0.32 *μ*g/ml [147]. Aqueous leaf extract caused a dose-dependent decrease in HeLa cell viability with IC_50_ of 70 *μ*g/mL [148]. Leaf extracts displayed significant antiproliferative activity (*p* < 0.05) against human liver (hepatocellular carcinoma, Hep-G2) and muscular (rhabdomyosarcoma, RD) cell lines [149]. The IC_50_ of leaf extracts cytotoxicity on cisplatin-resistant ovarian cancer (A2780CP20) and prostate cancer (PC3) cell lines in a study was 0.27 and 0.17 mg/mL, respectively [150]
		Apoptosis assay performed using leaf and bark extracts on breast and colorectal cancer lines showed a remarkable increase in the number of apoptotic cells with a sevenfold increase in breast (MD-MB-231) cell line to an increase of several folds in colorectal cancer (HCT-8) cell line [146]
		Leaf extracts inhibited the growth of hepatocarcinoma (HepG2), colorectal adenocarcinoma (Caco-2), and breast adenocarcinoma (MCF-7) cell lines with dichloromethane leaf extract having IC_50_ between 112 and 133 *μ*g/ml [151]. Leaf extracts caused the death of 72–82% of acute myeloid leukemia cells and 77–86% of acute lymphoblastic leukemia cells after 24 hours of incubation with 20 *μ*g/ml of the extract. At the same time, 69–81% of HepG2 cells died after treatment with ethanol extract [152]. Leaf extracts also showed *in vitro* anticancer activity on human hepatocellular carcinoma (HepG2) cells. At a maximum dose of 200 mg/kg, the survival of HepG2 and non-small-cell lung cancer (A549) cells was reported to decrease by 60% and 50%, respectively [153]
		Leaf extract had anticancer activity against human epidermoid cancer (Hep2) cell line with IC_50_ of 12.5 *μ*g/mL in the most active fraction [154]. Cytotoxicity of water-soluble leaf extract was reported against human alveolar epithelial cells derived from the lung cancer (A549) cell line with IC_50_ of 166.7 *μ*g/mL [155]
		Cell viability of leaf extract-treated A549, HepG2, CaCo2, Hek293, and Jurkat cells was reported to be reduced with IC_50_ from 0.05 to 0.4% [156]
		Human pancreatic cancer cells (Panc-1, p34, and COLO-357) were inhibited by leaf extracts with IC_50_ of 1.1, 1.5, and 1.8 mg/mL [157]
		Seed extracts had cytotoxic potential against A549, Hep-2, HT-29, and IMR-32 cancer cell lines [158]. *β*-sitosterol-3-oglucopyranoside, 4-(*α*-L-rhamnosyloxy) benzyl isothiocyanate, and niazimicin prevented the induction of Epstein–Barr virus genome in Raji cells. Niazimicin delayed the formation of tumors and reduced the number of tumors *in vivo* [159]

*Opuntia* species	Quercetin, kaempferol-3-O-rutinoside, isorhamnetin-3-O-rutinoside, betanin, and indicaxanthin [160]	Fruits, peels, seed, cladode, stem, and root extracts of different species have cytotoxicity against mammary (MCF-7), prostate (PC3), colon (Caco2, SW-480, HT-29), HeLa cervical carcinoma, myeloid leukemia (K562), and hepatic (HepG2) cell lines [161–164]
*Oxalis corniculata* Linn.	Palmitic, 8 oleic, linoleic, linolenic, stearic, tartaric, and citric acids, flavones (acacetin and 7,4′-diOMe apigenin), glycoflavones (4′-OMe vitexin, 4′-OMeiso-vitexin and 3′,4′-diOMe orientin), flavonols (3′,4′-diOMe quercetin), and phenolic acids such as *p*-hydroxybenzoic, vanillic, and syringic acids [165]	The ethanolic extract inhibited tumor growth of Ehrlich ascites carcinoma (EAC) induced in Swiss albino mice [166]

*Prunus africana* (Hook.f.) Kalkman	Ursolic acid, oleanolic acid, *β*-amyrin, atraric acid, *N*-butylbenzene-sulfonamide, *β*-sitosterol, *β*-sitosterol-3-O-glucoside, ferulic acid, and lauric acid [167–169]	Antiprostate cancer activity targets fast dividing cells by impairing mitosis or by causing target cells to undergo apoptosis [169, 170]. There was growth inhibition of a human prostate cancer cell line (PC-3) and epithelial cells derived from a lymph-node carcinoma of the prostate (LNCaP) by 50% at 2.5 *μ*L/mL and also induced significant apoptosis in both cell lines (PC-3 and LNCaP) at 2.5 *μ*L/mL compared to untreated cells. The ethanolic extract had an antimitogenic effect on prostate cancer cells by inhibiting the mitogenic action of epidermal growth factor which resulted in a decreased number of cells entering the S-phase of the cell cycle [171]

*Zanthoxylum chalybeum* Engl.	Skimmianine, furoquinoline alkaloid skimmianine, the benzophenanthridine alkaloids chelerythrine and nitidine, the aporphine alkaloids tembetarine, magnoflorine, N-methylcorydine, *N*-methylisocorydine (menisperine), and berberine and the phenylethylamine candicine, alkamide, fagaramide, dihydrochelerythrine, lupeol, and sesamin [172]	Extracts showed moderate cytotoxicity with IC_50_ values below 50 *μ*M against the drug-sensitive CCRF-CEM and multidrug-resistant CEM/ADR5000 leukemia cell lines [172]
		Stem bark extracts exhibited potential cytotoxicity effect with LC_50_ value of 5.74 *μ*g/mL in brine shrimp assay [173]
		Cytotoxicity reported against human cancer cell line HL-60 cells with IC_50_ of 137.31 *μ*g/mL and selectivity index of 3.81 [174]. Cytotoxicity against human gingival fibroblasts cells with IC_50_ of 26 ± 3 *μ*g/mL [175]
		Cytotoxicity of root bark extracts reported with IC_50_ of 38.5, 68.9 and < 500 *μ*g/mL in brine shrimp toxicity assay [97, 134, 176]

IC_50_: -median inhibitory concentration/half maximal inhibitory concentration, LC_50_: median lethal concentration, and IC_90_: concentration inhibiting 90% of cellular growth.

**Table 3:** Ethnomedicinal uses and other biological activities of the anticancer plant species reported in Uganda as per global studies.

Plant	Ethnomedicinal uses	Biological activities
*Abelmoschus esculentus* (L.) Moench	Treatment of syphilis, immunity boosting and treatment of anaemia, cuts, wounds, boils, catarrhal infections, ardor urinae, dysuria, and gonorrhoea [177]	Immunomodulating, antioxidant, antidiabetic, and antihyperlipidemic activities [178]
*Albizia coriaria* (Welw. Ex Oliver)	Treatment of heart diseases, meat allergy, nausea, headaches, mental illness, diarrhea, cough, tuberculosis, anaemia, syphilis, postpartum haemorrhage, snakebites, sore throats, herpes zoster, menorrhagia, stimulating milk production in lactating mothers, threatened abortion, skin diseases, jaundice, cough, steam fumigation treatments for sore eyes, and as a general tonic [33, 142, 179]	Antibacterial activity [58]
*Allium sativum* L.	Arteriosclerosis, management of diabetes mellitus, asthma, deafness, leprosy, bronchial congestion, fevers, worms, and liver gall bladder trouble [180]	Antidiabetic, hepatoprotective, antimicrobial, and antihyperlipidemic activities [181]
*Annona muricata* L.	Leaves are used to treat cystitis, diabetes, headaches, colds, flu, asthma, and insomnia [182]	Antiviral, antinociceptive, anti-inflammatory, and antihyperglycemic activities [183]
*Artemisia annua* L.	Treatment of cough, indigestion, malaria, fever caused by tuberculosis, and jaundice [33]	Antimalarial, antimicrobial, and anti-inflammatory activities [184]
*Beta vulgaris* L.	Management of hypertension, purifying blood and liver, and reducing inflammation [32]	Antioxidant and antibacterial activities [94]
*Blighia unijugata* Baker	Treating fibroids [43], vomiting, malaria, and skin diseases [185]	Antiplasmodial, antimalarial, and antioxidant activities [186]
*Opuntia* species	Treatment of skin wounds, stomach swelling, digestive problems, type 2 diabetes, colitis, high cholesterol, urinary tract infections, weight loss aid to treat obesity and overweight, and as a remedy for alcohol hangovers [40, 41, 187]	Antidiabetic, antimicrobial, antioxidant, and anti-inflammatory activities [188]
*Cannabis sativa* L.	Treatment of cough, tuberculosis, cancer pain, asthma and diarrhea [33], appetite stimulant in debilitating diseases, attenuation of wasting [189], antiemetics, treatment of multiple sclerosis, spinal cord injuries, Tourette's syndrome, epilepsy, and glaucoma [190]	Anti-inflammatory and antinociceptive activities [191]
*Canarium schweinfurthii* Engl.	Management of anaemia [39], dysentery, coughs, chest pains, tuberculosis, stomach complaints, food poisoning, purgative and emetic, roundworm infections and other intestinal parasites. Used as an emollient, stimulant, diuretic and in treatment of skin infections, eczema, leprosy, ulcers, diabetes mellitus, colic, stomach pains, pains after childbirth, gale, inappetence, fever, constipation, malaria, sexually transmitted infection, and rheumatism [33, 192, 193]	Antioxidant, analgesic, antibacterial, anti-inflammatory, and antidiabetic activities [194, 195]
*Carica papaya* L.	Treatment of cough, diarrhea, snake bite, sterility, pain killer, antidotes, promotes labour, cracks on soles of feet, low immunity, loss of memory, measles, erectile dysfunction [42, 43], and fevers [196], leaves used as an antipyretic, for malaria, splenomegaly, and HIV treatment [33, 185, 197, 198]. Fruit used to manage anaemia, worms, asthma, and tonsillitis [199]. Root extracts with that of *Carissa edulis* and *Euclea divinorum* used for treatment of venereal diseases [200]	Antibacterial, antimalarial, antifungal, and immunomodulatory activities [106, 107, 201]
*Capsicum frutescens* L.	Management of hernia, pancreas disorders, erectile dysfunction (roots) [43]. Used with *Clematis hirsuta* Perr. & Guill. for treating influenza and mental problems [202], headache, and indigestion [199]. As a salve to relieve muscle, joint, and toothache pain, to treat cough, asthma, and sore throat, as a stimulant, and to treat stomachache, seasickness, and flatulence [203]	Insulinotropic activities [204]
*Catharanthus roseus* (L.) G. Don.	Treatment of diabetes, malaria, dengue fever, dysentery, insect bites, skin infection, diarrhea, leukemia, eye irritation, dyspepsia, dysentery, toothache, sore throat, and lung congestion [205–207]	Anti-inflammatory, antibacterial, antifungal, antidiabetic, antihypercholesterolemic, antiandrogenic, and antiangiogenic activities [208]
*Citrus reticulata* Blanco, 1837	Treatment of skin diseases, malaria, and inducing weight loss [42]	Antimalarial and antioxidant activities [121, 122]
*Cymbopogon citratus* (DC) Stapf	Treatment of cough, fever, indigestion, pain in fallopian tubes [42, 43], malaria, and dental caries [185]	Antimalarial activity [125]
*Daucus carota* L.	Treating skin diseases and wounds [34], fatigue [33], cough, diarrhea, dysentery, malaria, as an antiseptic, abortifacient, aphrodisiac, carminative, stimulant, stomachic, and tonic [209]	Antioxidant and hepatoprotective activities [127]
*Elaeodendron buchananii (*Loes) Loes.	Treatment of wounds, syphilis, cough, and dysentery [210], blocked fallopian tube [43], candida [211], and urinary tract infections [212]	Antimicrobial, antioxidant and antifungal activities [211]
*Erythrina abyssinica* Lam. ex DC.	Treatment of viral infections [133], yellow fever, convulsions, anaemia, infertility in women, hiccup, vomiting, and urinary tract infections [212]	Antifungal activity [133]
*Euclea natalensis* A. DC.	Remedy for chest ailments, toothache, bronchitis, pleurisy, asthma, headache, and urinary tract infections [213, 214]	Antiplasmodial, antioxidant, antidiabetic, and antibacterial activities [215]
*Ficus dawei* Hutch.	Treating wounds [43]	NR
*Ficus natalensis* Hochst.	Treatment of HIV [198], diarrhea, vomiting, snakebites [33, 44], and heart diseases [179]	NR
*Lovoa trichilioides* Harms	Management of skin lesions, infections, and diarrhea [33]	NR
*Kigelia africana* Lam. Benth.	Treatment of wounds [35], skin diseases, eczema, psoriasis and leprosy [36], rheumatism, snakebites, and syphilis [216]	Antibacterial, antifungal, anti-inflammatory, analgesic, antidiabetic, and antioxidant activities [217]
*Markhamia lutea* (Benth.) K. Schum.	Management of anaemia, liver disease, inappetence, stomachache, headache, skin rash, cataracts, throat diseases, conjunctivitis, backache, and snakebites [33, 210, 218]	Antioxidant, antileishmanial, and antitrypanosomal activities [143, 144, 219]
*Moringa oleifera* Lam.	Cleansing blood and liver, strengthening the heart, increasing fat metabolism to promote weight loss, deworming, improving wound healing, reducing wrinkles, improving digestion, eliminating constipation, and body detoxification [45, 220]	Potent antioxidant, cardioprotective, antibacterial, hepatoprotective, antihypertensive, antiulcer, and anti-inflammatory activities [221, 222]
*Oxalis corniculata* L.	Treatment of athletes foot, wounds, hypertension, diabetes and hormonal imbalance [43], stomachache and migraine [44], excessive menstruation, cough, and antidote to poisoning [199]	Antifungal and antioxidative activities [223]
*Prunus africana* (Hook.f.) Kalkman	Used for managing fainting [43], malaria, HIV, cough, chest pain, epilepsy, heart diseases, diarrhea [33, 198], urinary tract infections, pregnancy [44], fever, and inappetence [102]	Anti-inflammatory, antioxidant, and antimalarial activities [215, 224]
*Vitex fischeri* Gürke	Treatment of herpes zoster, skin infections, rashes, tuberculosis [33]	Anticandidal activity [211, 225]
*Zanthoxylum chalybeum* Engl.	Treatment of stomachache, cough, fever, skin rush, diabetes [33, 42, 43], HIV [198], malaria, diarrhea, sickle cell, tuberculosis, pneumonia, colds, ulcers, sore throat, measles, bilharzia, amoebiasis, female sterility, uterine fibroids, and headache [226–230]	Anticandidal, antibacterial, and antidiabetic activities [173, 231–233]

NR: none retrieved in the open literature.
